# Non-negative matrix factorisation is the most appropriate method for extraction of muscle synergies in walking and running

**DOI:** 10.1038/s41598-020-65257-w

**Published:** 2020-05-19

**Authors:** Mohammad Fazle Rabbi, Claudio Pizzolato, David G. Lloyd, Chris P. Carty, Daniel Devaprakash, Laura E. Diamond

**Affiliations:** 1 School of Allied Health Sciences, Griffith University, Gold Coast, QLD 4222 Australia; 2 Griffith Centre of Biomedical and Rehabilitation Engineering (GCORE), Menzies Health Institute Queensland, Gold Coast, QLD 4222 Australia; 3 Department of Orthopaedic Surgery, Children’s Health Queensland Hospital and Health Service, Brisbane, QLD 4101 Australia; 4 Research Development Unit, Caboolture and Kilcoy Hospitals, Metro North Hospital and Health Service, Brisbane, QLD 4101 Australia

**Keywords:** Biomedical engineering, Computational neuroscience

## Abstract

Muscle synergies provide a simple description of a complex motor control mechanism. Synergies are extracted from muscle activation patterns using factorisation methods. Despite the availability of several factorisation methods in the literature, the most appropriate method for muscle synergy extraction is currently unknown. In this study, we compared four muscle synergy extraction methods: non-negative matrix factorisation, principal component analysis, independent component analysis, and factor analysis. Probability distribution of muscle activation patterns were compared with the probability distribution of synergy excitation primitives obtained from the four factorisation methods. Muscle synergies extracted using non-negative matrix factorisation best matched the probability distribution of muscle activation patterns across different walking and running speeds. Non-negative matrix factorisation also best tracked changes in muscle activation patterns compared to the other factorisation methods. Our results suggest that non-negative matrix factorisation is the best factorisation method for identifying muscle synergies in dynamic tasks with different levels of muscle contraction.

## Introduction

Despite decades of research, it remains unclear how the human central nervous system (CNS) coordinates activity of a large number of muscles during movement. Numerous studies suggest that the CNS activates muscles in groups to reduce the complexity required to control each individual muscle when performing a movement^1,2^. The coordination of muscles activated in synchrony is commonly referred to as muscle synergy^3^. Indirect evidence suggests that muscle synergies reside in the brainstem and/or spinal cord and follow a modular organization^4–6^. Muscle synergies are regarded as low dimensional units that produce complex activation patterns for a group of muscles, typically recorded via electromyography (EMG), during performance of a task^4,6^. Synergies can be observed at cortical^7^ or spinal^8^ levels, suggesting a high degree of cooperation within the CNS’s structural hierarchy^9^. Understanding the organisation of muscle synergies may help elucidate the neurological mechanisms that underpin a multitude of neurological conditions, including stroke^10–12^, cerebral palsy^13,14^, spinal cord injury^15^, and Parkinson’s Disease^16^.

Factorisation methods use recorded and processed EMG signals, from here referred to as muscle activation patterns, to quantify muscle synergies. A number of different factorisation methods have been used to extract muscle synergies from muscle activation patterns during dynamic tasks. The four most commonly used factorisation methods reported in the literature between 1999–2018 are non-negative matrix factorisation (NMF)^13,14,17–20^ (62.28%), principal component analysis (PCA)^21–24^ (23.11%), independent component analysis (ICA)^25,26^ (3.22%), and factor analysis (FA)^27,28^ (2.15%) (Literature search in Supplementary material). Applying a factorisation method to a set of muscle activation patterns yields two components: (i) synergy weights; and (ii) synergy excitation primitives. The synergy weights scale the neural command intensity of the excitation primitives to represent the time varying neural command^29^. Each factorisation method makes a different assumption regarding the variance of input data and employs a different algorithm to extract the muscle synergies. The NMF algorithm is implemented using the multiplicative update rule based on Euclidian distance objective function^17^, gradient descent^30^ and least square^31^ methods, and can be applied to both Gaussian and non-Gaussian datasets. NMF constrains muscle synergies to be non-negative and utilises second order statistics to find vectors that best describe the data’s variance. PCA extracts the muscle synergies that best describe the data’s variance while minimising covariance of the basis vectors (i.e. muscle synergy weights), and works best with Gaussian distributed datasets^29^. The basis vector minimisation is solved analytically using singular value decomposition, returning the eigenvectors of the data’s covariance matrix as the PCA identified muscle synergy weights. ICA is designed to deal with non-Gaussian variation in datasets and finds basis vectors (i.e. muscle synergy weights) that maximise the absolute value of the fourth moment of the data (i.e. kurtosis). Kurtosis is a measure of tailed-ness of the probability distribution of a real-valued random variable^32^ (indicating non-Gaussian data). Using pre-whitening, ICA transforms the data orthogonally to make it statistically independent^29,33^. Apart from these methods, other algorithms also implement ICA, including Infomax ICA (maximizes Shannon mutual information)^34^ and Kernel ICA (optimize variance to implement kernel Hilbert space)^35^. Similar to PCA, FA employs eigenvalue decomposition to produce eigenvectors (i.e. muscle synergy weights) of the covariance matrix. Extracted muscle synergies with eigenvalues>1 are considered significant and all synergies with eigenvalues <1 are considered noise^36^. All four factorisation methods use second or higher order statistics to estimate the muscle synergies from muscle activation patterns.

The performance of a factorisation method is highly dependent on variance in the recorded EMG signals. Variance accounted for (VAF) is typically employed to quantify reconstruction accuracy^30^, whereby muscle activation patterns are reconstructed by linearly combining synergy weights and excitation primitives. VAF statistically compares experimental and reconstructed muscle activation patterns, but does not provide any insight regarding the magnitude of neural information conveyed by the recorded EMG signal. Alternatively, the probability distribution of a signal, quantified by the probability density function (PDF), provides EMG spectral information and has a direct relationship with the neural and peripheral information conveyed by the signal itself^37,38^. Since different factorisation methods make different assumptions regarding the distribution of the muscle activation patterns, the probability distribution of the recorded EMG signal may indeed influence factorisation performance^30^. Previous research has demonstrated that the probability distribution of a recorded EMG signal can be characterised by a Gaussian during high and medium intensity isometric muscle contractions, and by a super Gaussian (Laplacian) function during low intensity isometric muscle contractions^32,39,40^. However, the probability distribution of recorded EMG signals from lower limb muscles during dynamic tasks such as walking and running is yet to be investigated.

NMF is the most commonly used factorisation method for muscle synergy analysis (Literature search in Supplementary material), perhaps because it is readily available in many commercial data processing packages (e.g. MATLAB). However, there is no clear evidence to support the use of NMF in place of other factorisation methods. Previous studies that compared factorisation methods primarily considered synthetic^30,41^, animal (e.g., frog^30^, cat^23^), and upper limb^29^ EMG datasets. Notably, Ivanenko and colleagues identified lower limb muscle synergies in humans during various walking conditions which remained stable when different factorisation methods were used^42^. However, none of these aforementioned studies compared factorisation methods for extraction of lower limb muscle synergies across both walking and running conditions. Furthermore, the probability distribution of included muscle activation patterns, an essential assumption of every factorisation method, has not previously been utilised as a metric for comparison. Therefore, this study aimed to use a PDF-based metric, along with VAF, to compare performance of the four most commonly used factorisation methods (NMF, PCA, ICA, FA) for extraction of lower limb muscle synergies during walking and running at different speeds. Given that NMF is the only factorisation method that makes no assumptions regarding distribution of the muscle activation patterns (i.e. Gaussian, non-Gaussian), we hypothesised that muscle synergies extracted using NMF would best capture the variation in PDF of muscle activation patterns during walking and running. Accordingly, we anticipated that muscle synergies extracted using NMF would best capture the variation of muscle activity due to gait speed and thereby better reconstruct the muscle activation patterns compared to the other factorisation methods.

## Methods

Eighteen healthy participants (72% male, age = 25.8 ± 6.1 years, height = 1.75 ± 0.76 m, mass = 64.9 ± 8.2 kg, body mass index = 21.1 ± 1.9 kg/m^2^) with no recurrent or recent lower limb injuries participated. This study was approved by the relevant institutional Human Research Ethics Committee of Griffith University (GU Ref no. 2017/020) and Australian Institute of Sport (Ref no. 20170801) and, all participants provided written informed consent prior to participation in the study. All methods were carried out in accordance with relevant guidelines and regulations of Helsinki.

### Data collection and processing

Surface EMG signals were recorded at 1500 Hz (Noraxon, Scottsdale, AZ, USA) from ten lower limb muscles: medial gastrocnemius, lateral gastrocnemius, soleus, peroneus longus, peroneus brevis, tibialis anterior, vastus lateralis, rectus femoris, biceps femoris, and sartorius. Skin preparation and electrode placement were performed according to SENIAM guidelines^43^. Participants completed ten trials of over-ground walking (1.3 ± 0.1 m/s) and running at slow (3 ± 0.3 m/s), moderate (5 ± 0.5 m/s), and fast (7 ± 0.7 m/s) speeds. Timing gates were used to ensure that average speed for each trial remained within 10% of the condition’s predefined limit.

Raw EMG signals were band-pass filtered at 30–400 Hz, full wave rectified, and low-pass filtered at 6 Hz using a zero-lag 4th order Butterworth filter to generate EMG linear envelops. For each trial (consisting of one full cycle, toe off to toe off), the linear envelops were amplitude normalised to the trial’s peak value^44^ and time normalised to 200 data points^45^, to ensure data from each muscle, trial, and task were equally represented in the muscle synergy analysis. Therefore, the processed EMG dataset (i.e. muscle activation patterns) computed from each trial consisted of one matrix with 10 rows (1 per muscle) and 200 columns (1 per time point).

### Muscle synergy analysis

Muscle activation patterns (*x*) can be reconstructed by multiplying the muscle synergy weights (*w*) and synergy excitation primitives (*h*):

1 $$x(t)=\mathop{\sum }\limits_{i=1}^{N}{w}_{i}{h}_{i}(t)+e(t)$$

where *e* represents reconstruction error. Equation (1) can be written in vector form as

2 $${\boldsymbol{x}}={\boldsymbol{wh}}+{\boldsymbol{e}}$$

where ***x*** is a *L* × *N* matrix representing the muscle activation patterns of *L* muscles at *N* time points, ***w*** is a *L* × *K* matrix containing time independent muscle weightings with *K* being the number of muscle synergies, and ***h*** is a *K* × *N* matrix of time varying synergy excitation primitives, and ***e*** is a *L* × *N* matrix representing the reconstruction error of each muscle’s activation pattern at each time point^46,47^. Reconstructed muscle activation patterns can be compared with experimental muscle activation patterns to evaluate the performance of the factorisation method used to identify the muscle synergies. In all four factorisation methods, muscle activation patterns are factorised by minimising the mean square error (MSE) between original and reconstructed muscle activation patterns. In general, the objective function can be written as $\mathop{{\rm{\min }}}\limits_{\widetilde{x}}\Vert {\boldsymbol{x}}-\widetilde{{\boldsymbol{x}}}\Vert$. In NMF $\widetilde{{\boldsymbol{x}}}={\boldsymbol{wh}}$ and ${w}_{i}\ge 0$; ${h}_{i}\ge 0$ is assumed for non-negativity^48^. In FA and PCA, $\widetilde{{\boldsymbol{x}}}={\boldsymbol{w}}{{\boldsymbol{h}}}^{{\boldsymbol{T}}}$ with ${{\boldsymbol{h}}}^{{\boldsymbol{T}}}{\boldsymbol{h}}{\boldsymbol{=}}{\boldsymbol{I}}$ assumed in PCA^49^. In ICA, $\widetilde{{\boldsymbol{x}}}={\boldsymbol{A}}{\boldsymbol{wh}}$ where ***A*** represents a mixing matrix which is used for pre-whitening of data^50,51^. Equation (2) can be re-written as

3 $${\boldsymbol{x}}=\widetilde{{\boldsymbol{x}}}+{\boldsymbol{e}}$$

To evaluate the variation in probability distribution of muscle activity at different walking/running speeds, we calculated the PDF of both the recorded (raw) EMG signals and the muscle activation patterns. Since the factorisation method is only applied to the muscle activation patterns and not to the raw EMG, the PDF of the muscle activation patterns was compared with the PDF of the excitation primitives. The average PDF for all recorded muscles followed a transition from a super Gaussian to Gaussian nature (Table 1). Thus, the reconstruction error can also be assumed Gaussian-distributed and variance of the error (${\sigma }_{e}^{2}$) can be used to calculate MSE as

4 $${MSE}={var}(\widetilde{{\boldsymbol{x}}})+{\sigma }_{e}^{2}$$

**Table 1:** Average (± standard deviation) probability density function kurtosis and skewness of raw EMG signals and muscle activation patterns from 10 muscles for 19 participants during walking and running at different speeds (10 trials each).

	Walk		Slow run		Moderate run		Fast run	
	Raw EMG	Muscle activation patterns	Raw EMG	Muscle activation patterns	Raw EMG	Muscle activation patterns	Raw EMG	Muscle activation patterns
Kurtosis	1.75(±0.19)	1.72(±0.20)	1.45(±0.21)	1.42(±0.20)	1.29(±0.19)	1.25(±0.18)	1.1(±0.2)	1.0(±0.10)
Skewness	0.01(±0.02)	0.012(±0.02)	0.009(±0.02)	0.012(±0.01)	0.005(±0.01)	0.01(±0.01)	0.003(±0.01)	0.009(±0.01)

FA: factor analysis; ICA: independent component analysis; NMF: non-negative matrix factorisation; PCA: principal component analysis.

If the distribution of ***x*** and ***h*** is the same (given the other parameter - muscle synergy weights (***w***) - is independent), then the error variance (${\sigma }_{e}^{2}$) is known. Also, given that ***w*** represents scalar weights that linearly scale and combine ***h*** to produce $\widetilde{{\boldsymbol{x}}}$, then the PDF of $\widetilde{{\boldsymbol{x}}}$ is given by PDF of ***h***. In other words, the best estimate of muscle activation ($\widetilde{{\boldsymbol{x}}}$) can be calculated when ***x*** and ***h*** have the same distribution. Thus, the similarity between probability distribution of ***x*** and ***h*** is a metric that can be used to compare factorisation methods.

### Comparison of factorisation methods

The PDF of the muscle activation patterns was modelled using a Gaussian probability distribution function

5 $$P(x)=\frac{1}{{\sigma }_{x}\sqrt{2\pi }}{e}^{\frac{{-(x-{\mu }_{x})}^{2}}{2{\sigma }_{x}^{2}}}$$

where ${\mu }_{x}$, and ${\sigma }_{x}$ represent the mean and standard deviation of muscle activation patterns, respectively. Similarly, the PDF of the synergy excitation primitives identified by the factorisation methods was calculated as

6 $$P(h)=\frac{1}{{\sigma }_{h}\sqrt{2\pi }}{e}^{\frac{{-(h-{\mu }_{h})}^{2}}{2{\sigma }_{h}^{2}}}$$

where ${\mu }_{h}$, and ${\sigma }_{h}$ represent the mean and standard deviation of excitation primitives, respectively.

The Kolmogorov-Smirnov (KS) test was used to compare the probability distribution of muscle activation patterns and synergy excitation primitives. The KS test is based on differences in both location and shape of the cumulative distribution functions of two samples regardless of the type of distributions and the number of samples^52^. The cumulative distributions of $P(x)$ and $P(h)$ was calculated using

7 $$F(x)={\int }_{-{\rm{\infty }}}^{x}P(x){dx}$$

8 $$F(h)={\int }_{-{\rm{\infty }}}^{x}P(h){dh}$$

The cumulative distributions represented by Eqs. (7) and (8) are equivalent to the empirical cumulative distribution function (ECDF)^53^, which estimates the cumulative distribution of a random variable by assigning equal probability to each observation in a sample. The ECDF allows for two distributions to be compared without knowing the exact probability distributions associated to ***x*** and ***h***. The KS test yields the maximum dissimilarity between the ECDF of ***x*** and ***h***, where the dissimilarity between $F(x)$ and $F(h)$ was calculated

9 $$D={\max }|F(x)-F(h)|$$

As the number of muscle synergies is always less than the number of muscles (i.e. *K* < *L*), there is a dimension mismatch between $F(x)$ and $F(h)$ in Eq. (9). To compare these two ECDFs, we assessed the similarity between the ECDF of each *k*^th^ synergy excitation primitive and all *L* muscle activation patterns by calculating maximum dissimilarities between two ECDFs as

10 $${D}_{k}=max|{F}_{l}(x)-{F}_{k}(h)|,\,l=1,\,2,\,\cdots ,\,L;\,k=1,\,2,\,\cdots ,\,K$$

**Figure 1:**
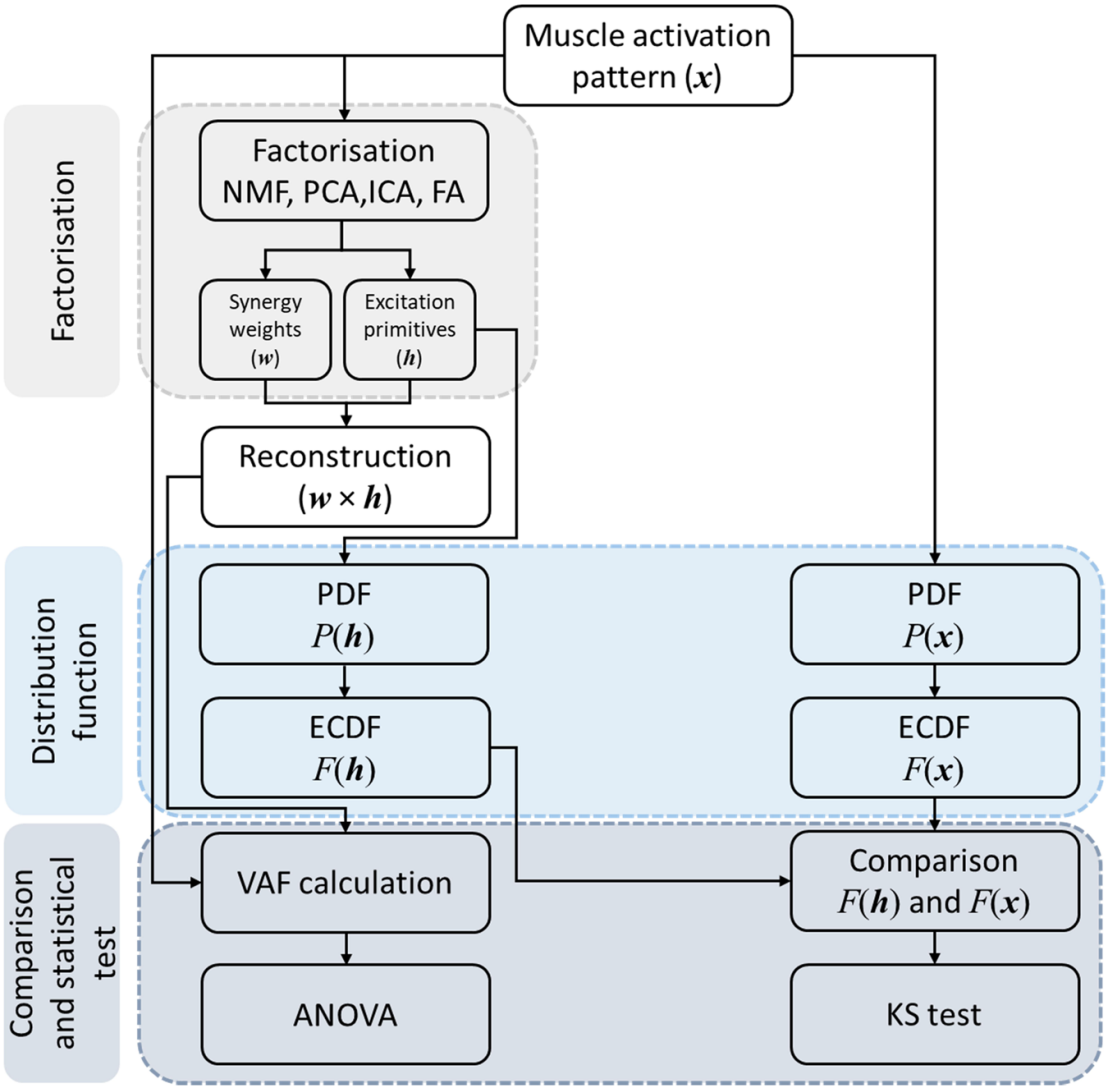
Workflow describing the methods used to compare the performance of four factorisation methods for muscle synergy analysis.

After calculating *D*_*k*_ using equation (10), the maximum dissimilarity index (*D*) was calculated as the maximum of *D*_*k*_ among all *k* as $D=\,\max |{D}_{k}|$. Maximum dissimilarity represents the single worst-case scenario of similarity between the ECDFs of the excitation primitives and muscle activation patterns. This workflow (Fig. 1) was repeated for all four factorisation methods. If the ECDFs of ***x*** and ***h*** were not hugely different, the KS test failed to reject the null hypothesis (i.e. significant differences between ECDFs occurred at p < 0.05)^54,55^. In addition to the maximum dissimilarity, percentage of occurrence of agreement (i.e. rate of failure to reject the null hypothesis at p < 0.05) was also used to evaluate the similarity between the probability distribution of muscle activation patterns and synergy excitation primitives.

The reconstruction performance of each factorisation method varies according to the number of muscle synergies used^30^. VAF can be calculated to determine the minimum number of muscle synergies needed to adequately reconstruct muscle activation patterns for each task

11 $$VAF=1-\frac{\Vert {({\boldsymbol{x}}-{\boldsymbol{w}}\ast {\boldsymbol{h}})}^{2}\Vert }{{\Vert {\boldsymbol{x}}\Vert }^{2}}$$

VAF was calculated for a number of synergies (*K*) between 2 and 6 for each of the four selected factorisation methods. A final value of *K* was selected with VAF greater than 85% for all factorisation methods. The minimum number of synergies was then used to compare factorisation methods using the KS test.

The factorisation methods were compared based on VAF between the original and reconstructed muscle activation patterns and PDF similarity was compared between the original muscle activation patterns and synergy excitation primitives (Fig. 1). The PDF similarity was assessed using occurrence of agreement and maximum dissimilarity index for each gait speed. The individual metrics for each factorisation method across all gait speeds were compared using a repeated measure analysis of variance (ANOVA) with Bonferroni correction.

## Results

**Figure 2:**
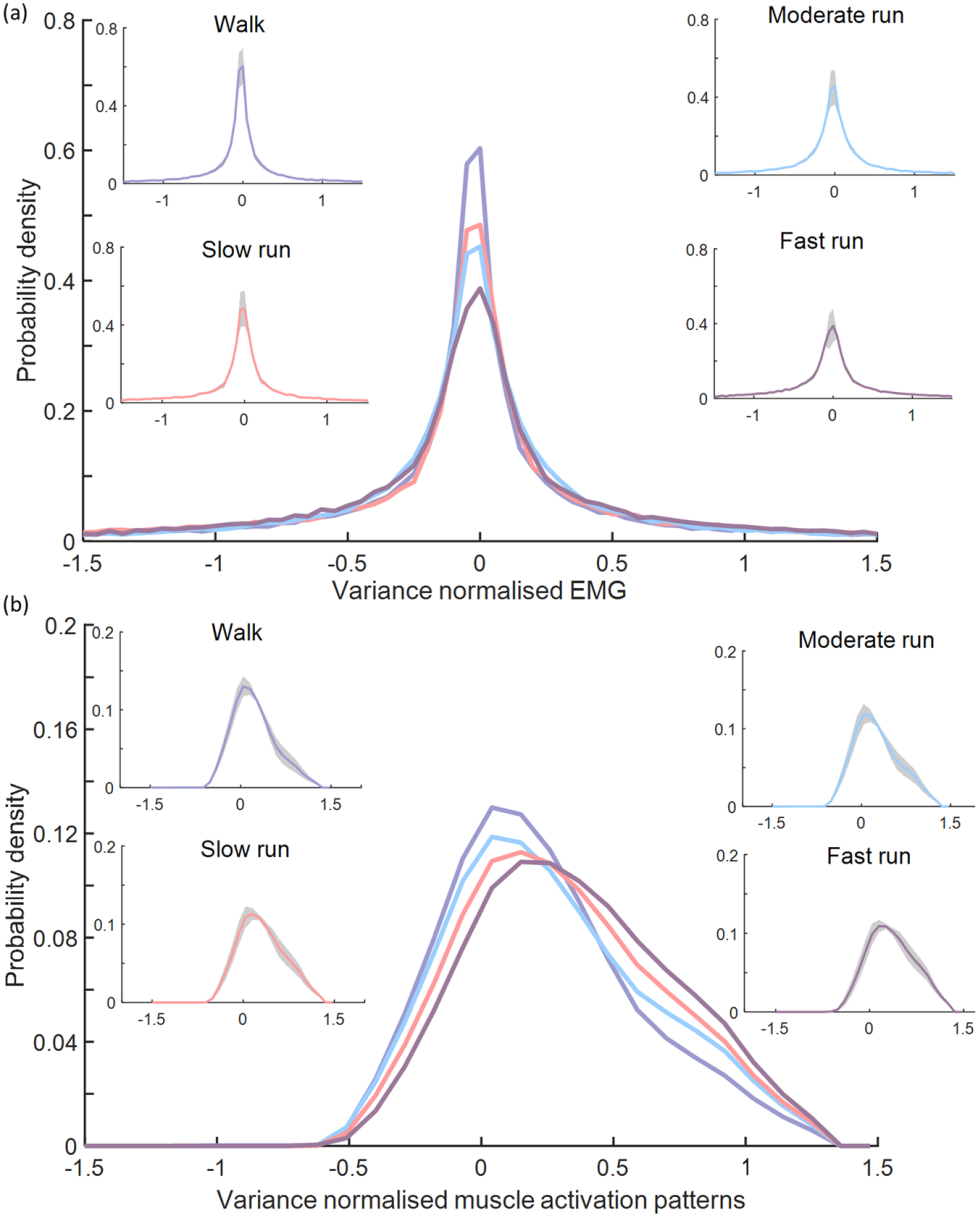
Average probability distribution of (**a**) raw electromyogram (EMG) signals and (**b**) muscle activation patterns from 10 lower limb muscles (medial gastrocnemius, lateral gastrocnemius, soleus, peroneus longus, peroneus brevis, tibialis anterior, vastus lateralis, rectus femoris, biceps femoris, and sartorius) from a representative participant during walking and slow, moderate, and fast running conditions. Shaded regions indicate standard deviation across 10 trials. The probability density function for both the raw EMG and muscle activation patterns widened as a function of increasing gait speed.

The mean PDF of the raw EMG signals and muscle activation patterns from 10 muscles from a representative participant are shown in Fig. 2, while the PDF and ECDF of raw EMG signals from individual muscles in all participants are reported in Supplementary Figs. S1 and S2, respectively. Average kurtosis of the PDFs across all participants decreased as gait speed increased, indicating that the PDF is transformed from Laplacian (theoretical kurtosis = 3)^32,56^ to Gaussian (theoretical kurtosis = 0)^32,56^ with increasing speed (Table 1). Minimal positive skewness of the PDFs was observed as gait speed increased, indicating high symmetry between the left and right side of the PDF^40^.

**Figure 3:**
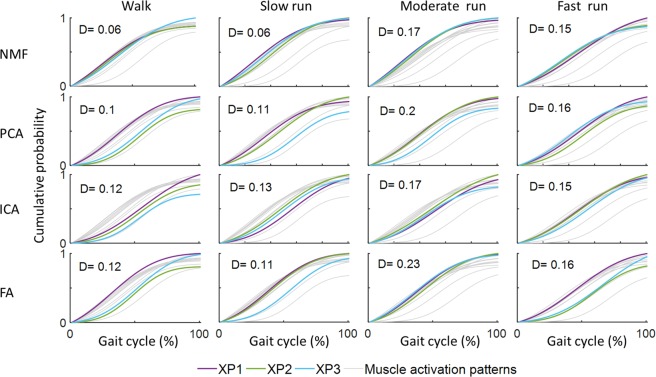
ECDF between muscle activation patterns and synergy excitation primitives, and corresponding maximum dissimilarities (D) (equation 10) for one representative participant. Lower maximum dissimilarity indicates greater similarity between ECDFs from the muscle activation patterns and synergy excitation primitives.

Extracting three muscle synergies (*k*=3) accounted for>85% of variance in muscle activation patterns for all factorisation methods. Typically, NMF generated ECDFs with greater similarity and lower maximum dissimilarities indices (*D*) between muscle activation patterns and synergy excitation primitives, compared to the other factorisation methods (Fig. 3).

**Figure 4:**
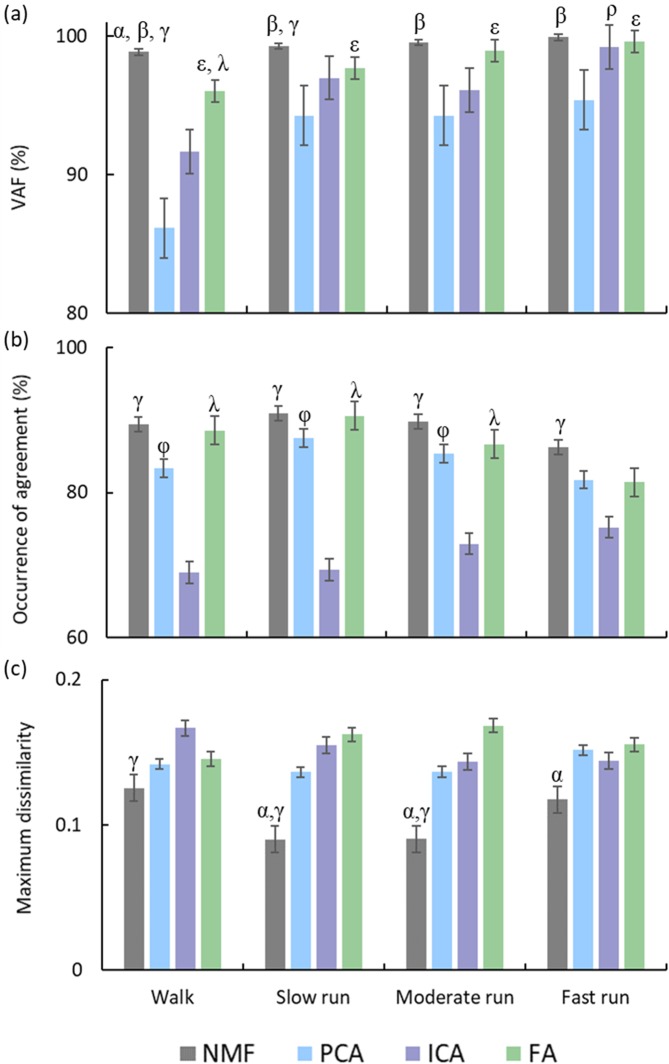
(**a**) VAF (%), (**b**) occurrence of agreement (%), and (**c**) maximum dissimilarity between the emperical cumulative distribution function (ECDF) of the muscle activation patterns and synergy excitation primitives at four gait speeds. Error bars indicate standard deviation. The following symbols represent significant differences (p < 0.05) between factorisation methods, α: NMF > FA or NMF < FA, β: NMF > PCA, γ: NMF > ICA or NMF < ICA, ε: FA > PCA, λ: FA > ICA, ρ: ICA > PCA, φ: PCA > ICA.

**Figure 5:**
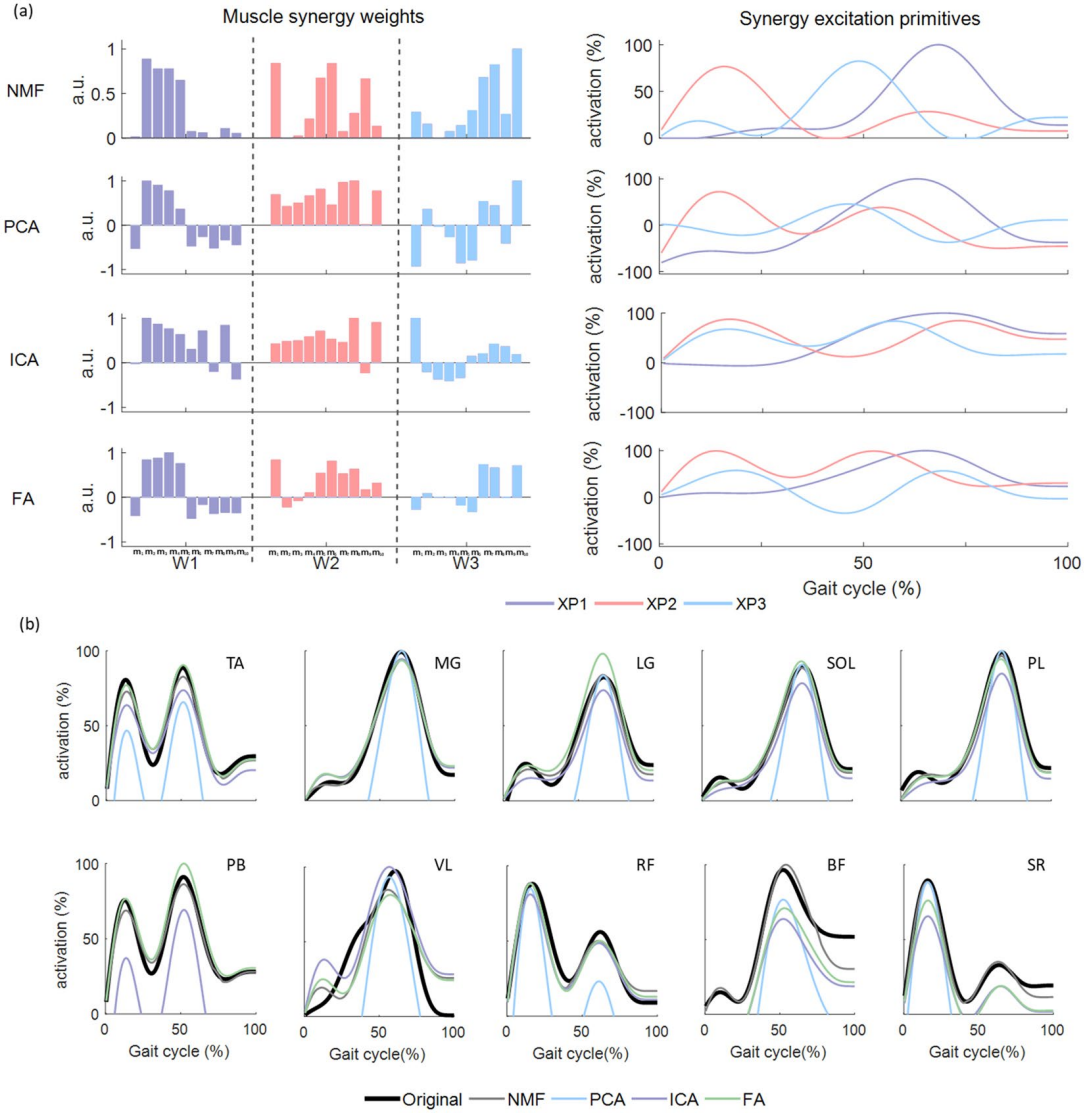
Reconstruction of muscle activation patterns with three muscle synergies using each factorisation method during walking for one representative participant: (**a**) muscle synergy weights (W_1_-W_3_) from 10 included muscles and synergy excitation primitives identified using NMF, PCA, ICA and FA, (**b**) original muscle activation patterns and reconstructed muscle activation patterns for 10 included muscles. a.u: arbitrary unit.; m_1_: biceps femoris; m_2_: lateral gastrocnemius; m_3_: medial gastrocnemius; m_4_: peroneus longus; m_5_: peroneus brevis; m_6_: rectus femoris: m_7_: sartorius; m_8_: soleus; m_9_: tibialis anterior; m_10_: vastus lateralis.

VAF was significantly higher using NMF than the other factorisation methods (p < 0.01) for walking. It was also higher than PCA and ICA (p < 0.05) for the slow run condition, and higher than PCA (p < 0.01) for the moderate and fast run conditions (Fig. 4a). NMF in general resulted in the highest occurrence of agreement of ECDF for all gait speed conditions (Fig. 4b), and was found to be significantly higher than PCA and ICA for walking (p < 0.01). PCA and FA had higher agreement than ICA for the slow and moderate run conditions (p < 0.01). In addition, NMF had the lowest dissimilarity among all factorisation methods for all speed conditions (Fig. 4c). The maximum dissimilarity of NMF was found to be significantly lower than ICA for walking and for slow and moderate run conditions (p < 0.01), and lower than FA for slow, moderate (p < 0.05), and fast (p < 0.01) run conditions. An example of reconstruction of muscle activation patterns using four factorisation methods for four gait speeds is reported in Fig. 5. Original and reconstructed muscle activation patterns of individual muscles from all participants for four gait speeds are shown in Supplementary Fig. S3.

## Discussion

This study aimed to use a PDF-based metric, along with VAF, to compare performance of the four most commonly used factorisation methods (NMF, PCA, ICA, FA) for extraction of lower limb muscle synergies during walking and running at different speeds. In agreement with our hypotheses, NMF demonstrated superior performance for all three metrics when three muscle synergies were extracted. These findings suggest that NMF may have unique ability to capture changes in the probability distribution of muscle activation patterns across different speeds, indicating that NMF is the best factorisation method for identifying muscle synergies in dynamic tasks with different levels of muscle contraction.

Our analysis identified that the PDF of the raw EMG signals from 10 lower limb muscles widened as a function of increasing gait speed. Notably, the probability distribution transitioned from Laplacian to Gaussian as gait speed increased, which is supported by a corresponding decrease in kurtosis and skewness. This transition is in line with previous investigations where similar changes in PDF of upper-limb EMG signals were observed with increasing isometric contraction intensity^39,40^, and in agreement with animal studies^57,58^. Given that the factorisation methods were applied to the muscle activation patterns and not directly to the raw EMG signals, we verified that the PDF of the muscle activation patterns demonstrated similar behaviour to the PDF of the raw EMG signals (Table 1; Fig. 2). As both PDFs vary as a function of gait speed, it is essential that a factorisation method capture these variations in order to reconstruct muscle activation patterns effectively. We examined the variation of PDF and ECDF of excitation primitives across four gait speeds (Supplementary Figs. S4 and S5). PDFs of the excitation primitives from all factorisation methods vary as a function of speed, except those obtained from FA (Supplementary Table T1). We also assessed whether the PDF of the muscle activation pattern was affected by the low-pass filter cut-off frequency used in creating the linear envelop. We evaluated the PDF using three different cut-off frequencies (10 Hz, 8 Hz and 6 Hz). Results demonstrate that the PDF of individual muscle activation patterns remain unaffected by cut-off frequency in this range (Supplementary Fig. S6).

Three metrics (VAF, occurrence of agreement, and maximum dissimilarity) were used to compare performance of NMF, PCA, ICA, and FA using three muscle synergies (Fig. 4). NMF performed significantly better than the other factorisation methods for at least one metric for each gait speed condition. When considering VAF alone, the factorisation methods performed equally for running; however, when PDF similarity was also taken into account, NMF outperformed the other factorisation methods. When we repeated our analysis using four muscle synergies, our primary metrics remained relatively unchanged (Supplementary Fig. S7). VAF is a statistical measure of signal reconstruction accuracy, while occurrence of agreement and maximum dissimilarity are used to compare the PDF of muscle activation patterns and synergy excitation primitives. Maximum dissimilarity was used as a goodness of fit to find the most similarity between the ECDFs of the excitation primitives and muscle activation patterns. Maximum dissimilarity will result in a high value even if the means of the two ECDFs are the same but the shape of the ECDFs are different (e.g. the tail of one ECDF is longer)^59^. Unlike VAF, the probability distribution (as assessed by occurrence of agreement and maximum dissimilarity) has a direct relationship with peripheral and neural information conveyed by the recorded EMG signal during any muscle activity^37,60^. Given the unique yet complimentary assessment parameters of VAF and PDF, implementing both metrics is likely to provide a more comprehensive understanding of reconstruction performance. Though we only considered walking and running at different speeds, it seems plausible that these results could be extrapolated to different dynamic tasks of varying intensities.

Our results demonstrate that NMF can capture the variation in PDF associated to speed-related changes in the raw EMG signal. As a direct consequence of this unique capability, the muscle synergies extracted using NMF provided the best representation of the muscle activation patterns recorded during gait at different speeds. In a previous study, muscle synergies extracted via NMF were shown to be similar across different gait (both walking and running) speeds when compared the muscle synergy weights^45^. As such, it may be possible to use only a single set of NMF identified muscle synergies to reconstruct the continuum of gait speeds, at least in healthy individuals. However, it is unclear whether our observations would be reproduced when investigating neurologically-impaired cohorts or different dynamic tasks.

This study has some limitations that warrant consideration. First, only healthy individuals were included and therefore these findings may not be generalisable to other populations. Nonetheless, since NMF does not rely on the type of distribution (Gaussian, non-Gaussian) of muscle activation patterns, it is likely to be the most sensitive method for characterising neuro-related alterations in the PDF. We utilised three muscle synergies (based on>85% VAF) for all four factorisation methods, which is in line with previous investigations using NMF^20,61^, PCA^23^, ICA^29^ and FA^27^ for walking and running at different speeds. Further, our primary metrics (VAF, occurrence of agreement, and maximum dissimilarity) remained relatively unchanged when four (Supplementary Fig. S7), five, six, seven, and eight synergies were extracted (data not presented).

NMF demonstrated superior performance for extraction of lower limb muscle synergies during walking and running at different speeds compared to PCA, ICA, and FA, based partly on our implementation of a PDF-based metric which identified that NMF can uniquely evolve with changes in gait speed. As such, NMF should be preferred for muscle synergy analysis applications that involve gait with variable speeds. Future investigations are required to confirm these findings in other dynamic tasks with varying intensities.

## Supplementary information

Supplementary Information.

## Data Availability

The datasets analysed in the current study is available from the corresponding author on responsible request.
