# Supplementary material for: Non-negative matrix factorisation is the most appropriate method for extraction of muscle synergies in walking and running
Source: Sci Rep. 2020 May 19;10:8266. doi: 10.1038/s41598-020-65257-w (PMC7237673; doi:10.1038/s41598-020-65257-w)
Supplement: Supplementary file 1 — Supplementary Information. [file 41598_2020_65257_MOESM1_ESM.docx]

**Non-negative matrix factorisation is the most appropriate method for extraction of muscle synergies in walking and running**

Mohammad Fazle Rabbi^1,2^, Claudio Pizzolato^1,2,*^, David G. Lloyd^1,2^, Chris P. Carty^1,2,3^, Daniel Devaprakash^1,2^, Laura E. Diamond^1,2^

^1^School of Allied Health Sciences, Griffith University, Gold Coast, QLD 4222, Australia

^2^Griffith Centre for Biomedical & Rehabilitation Engineering (GCORE), Menzies Health Institute Queensland, Gold Coast, QLD 4222, Australia

^3^Department of Orthopaedic Surgery, Children’s Health Queensland Hospital and Health Service, Brisbane, QLD 4101, Australia

^*^Corresponding author: c.pizzolato@griffith.edu.au

**Literature search**

A digital library and database (PubMed) was searched for literature related to muscle synergy on June 1, 2018. The keywords used in the search were “electromyography” OR "EMG” AND “synergy”. The search resulted in 485 documents, 186 of which analysed muscle synergies in different biomechanical tasks in healthy individuals and/or individuals with neurological conditions. A total of thirty-five studies analysed muscle synergies for assessment of ten neurological and musculoskeletal conditions (Supplementary figure 1a). The four most common neurological conditions resulted post-stroke, CP, SCI and Parkinson’s disease.


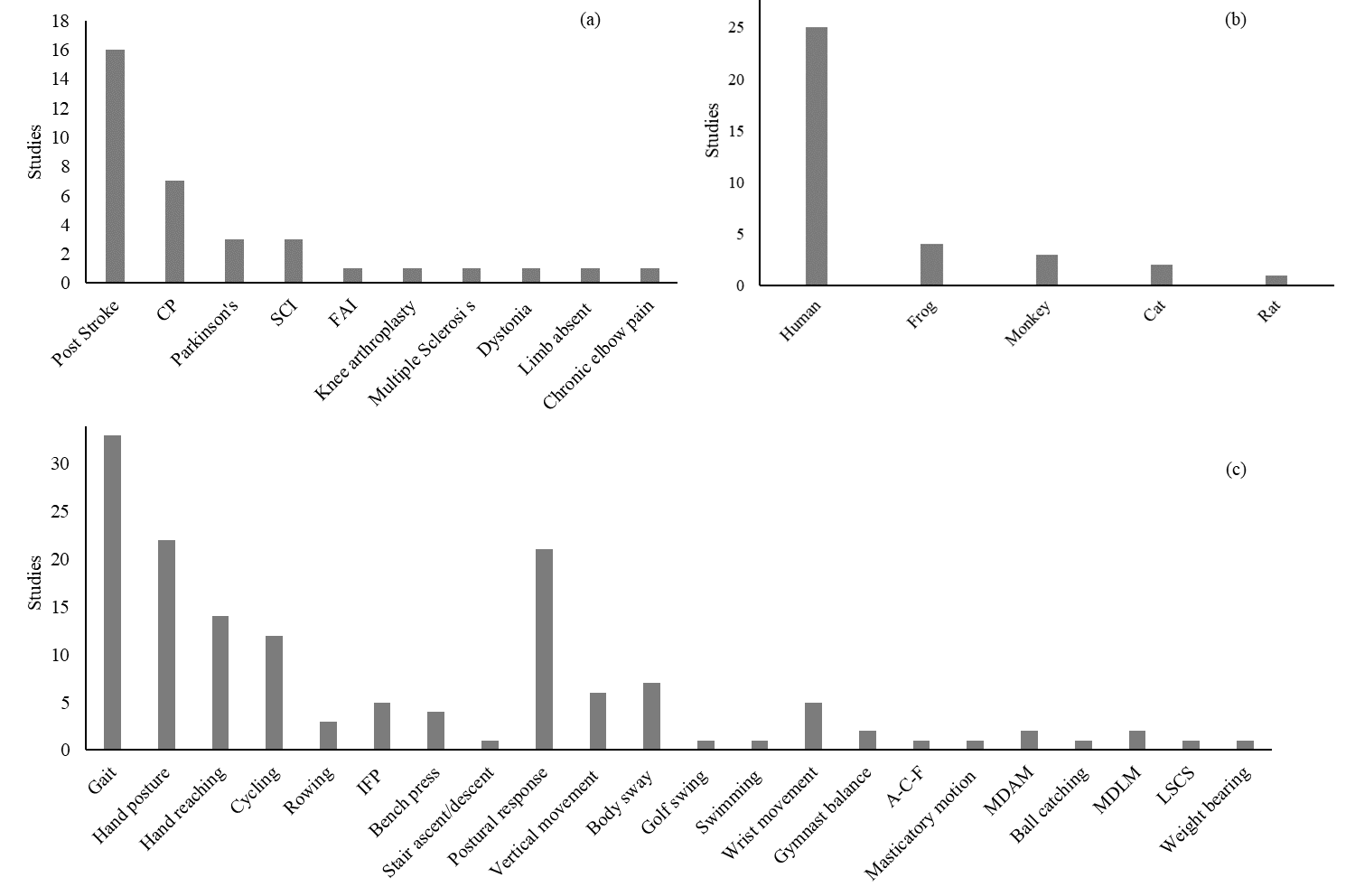


**Supplementary Figure L1.** (a) Neurological and musculoskeletal conditions, (b) types of subjects, (c) biomechanical tasks included in muscle synergy analysis in current literature. A-C-F: adduction-caudal extension-flexion; CP: cerebral palsy; FAI: femoroacetabular impingement; IFP: isometric force production (hand/leg); LE: lateral epicondylalgia; LSCS: lumbar spinal cord stimulation; MDAM: multi direction ankle movement; MDLM: multi direction leg movement; SCI: spinal cord injury.

The most common type of subject found in the literature was human (25 studies) followed by frog, monkey, cat and rat (Supplementary figure 1b). Additionally, a variety of biomechanical tasks were performed during EMG acquisition (Supplementary figure 1c). The most commonly performed tasks used for muscle synergy analysis were gait (walking and running), hand posture, postural response, hand reaching task and cycling, as found in 146 studies. 68.28% of the studies that conducted muscle synergy analysis used non-negative matrix factorisation (NMF) to identify muscle synergies during motor tasks (Supplementary figure 2). Other commonly used factorisation methods were principal component analysis (PCA) (23.11%) followed by independent component analysis (ICA) (3.22%) and factor analysis (FA) (2.15%) (Figure 2).


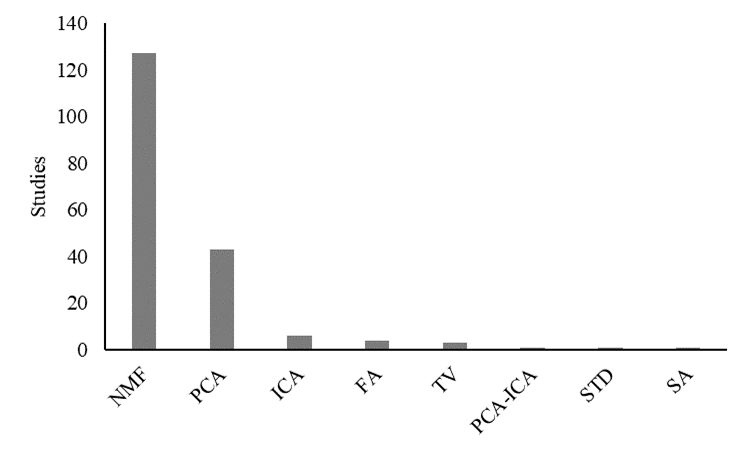


**Supplementary Figure L2.** Factorisation methods used for muscle synergy extraction in current literature. ICA: independent component analysis; FA: factor analysis; NMF: non-negative matrix factorisation; PCA: principal component analysis; SA: statistical analysis; STD: space-time decomposition; TV: time varying.

**Probability density function of raw EMG signals**


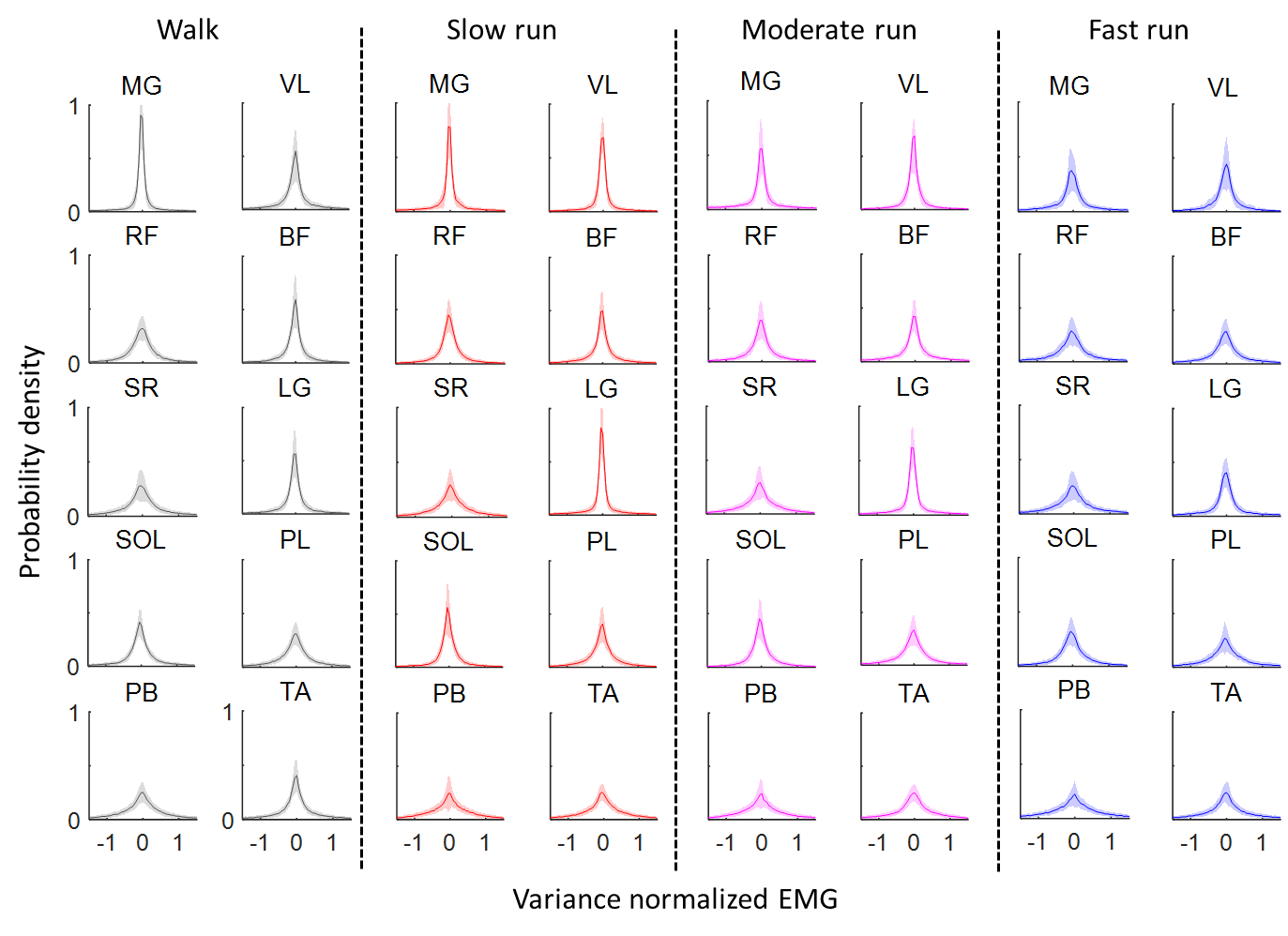


**Supplementary Figure S1.** Ensemble average of probability distribution of raw EMG signals from 10 lower limb muscles recorded from all participants during walking and slow, moderate, and fast running conditions. Shaded regions indicate standard deviation across participants.

**Empirical cumulative distribution of raw EMG signals**


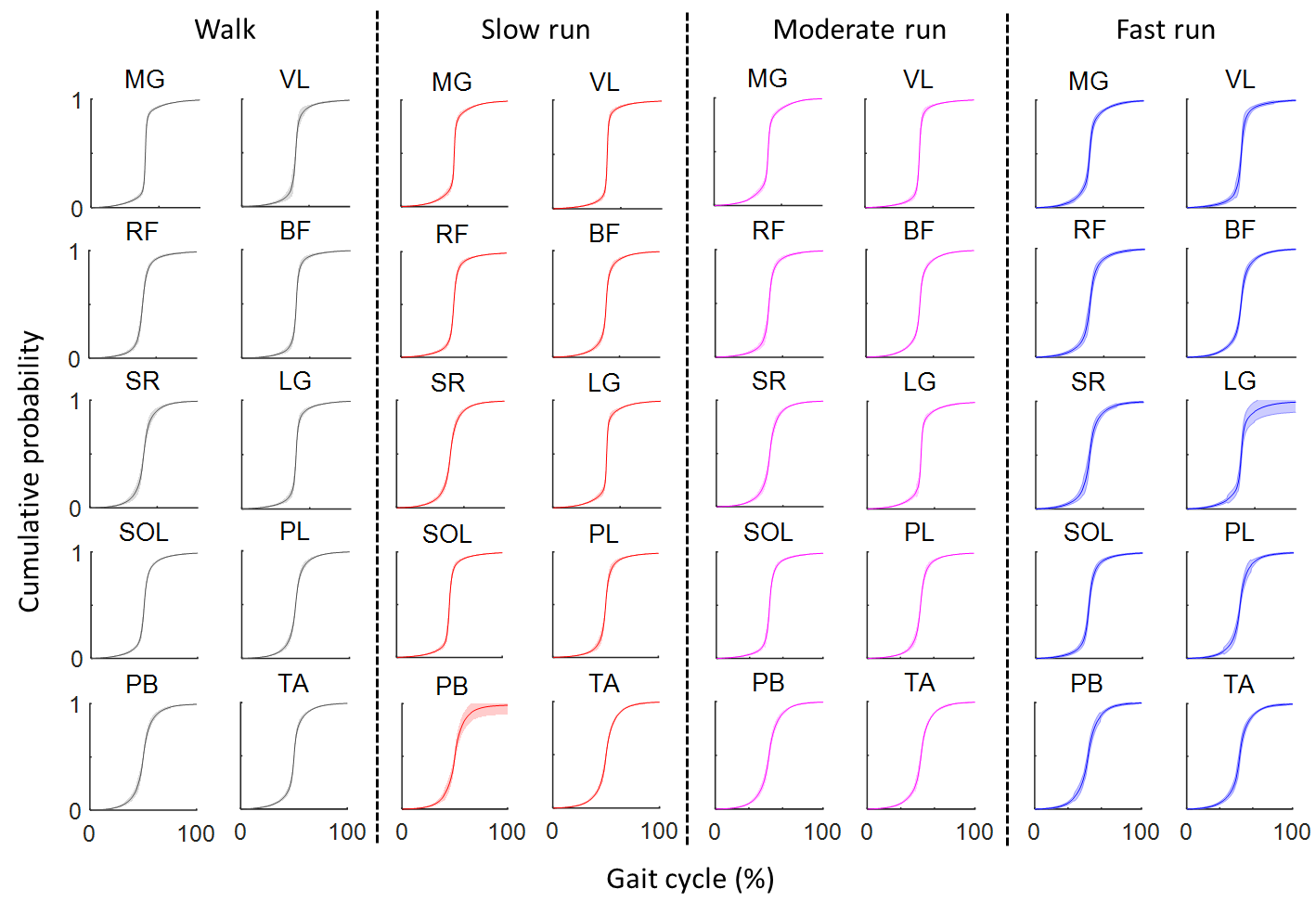


**Supplementary Figure S2.** Emperical cumulative distribution of raw EMG signals from 10 muscles of 18 participants during walking and slow, moderate, and fast running conditions. Shaded regions indicate standard deviation across participants.

**Original and reconstructed muscle activation patterns**


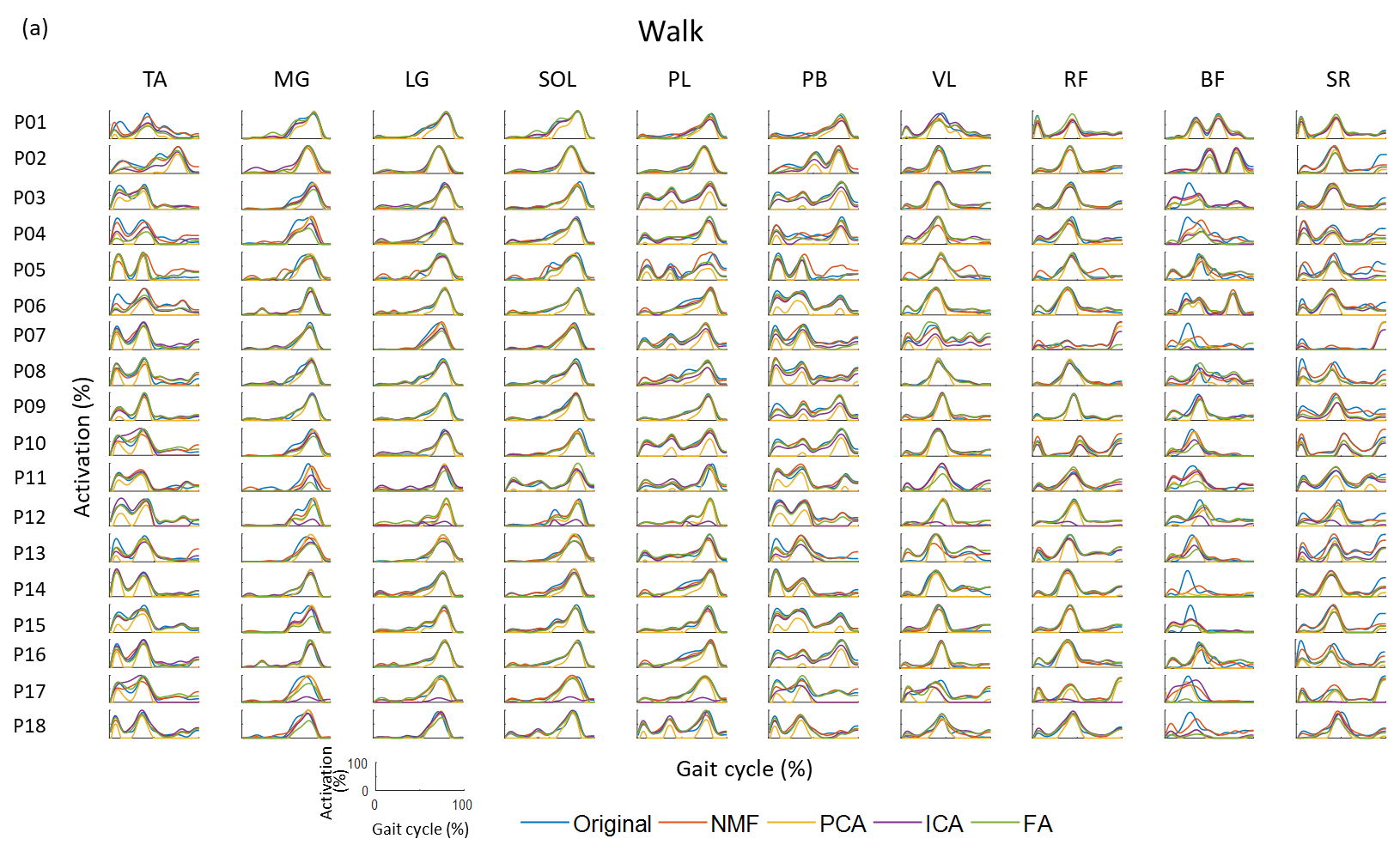


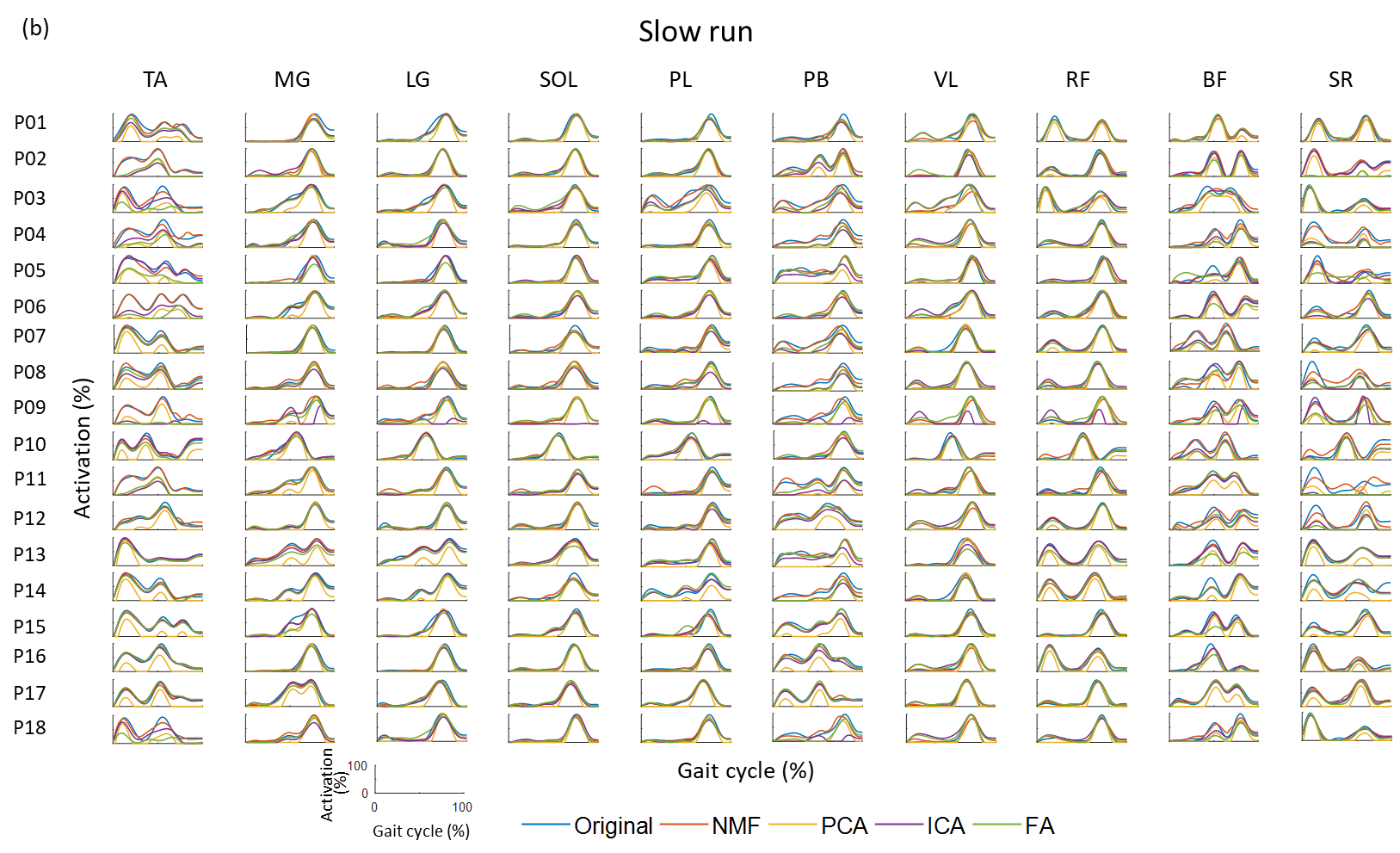


(b)

Supplementary Figure S3 (Cont.)


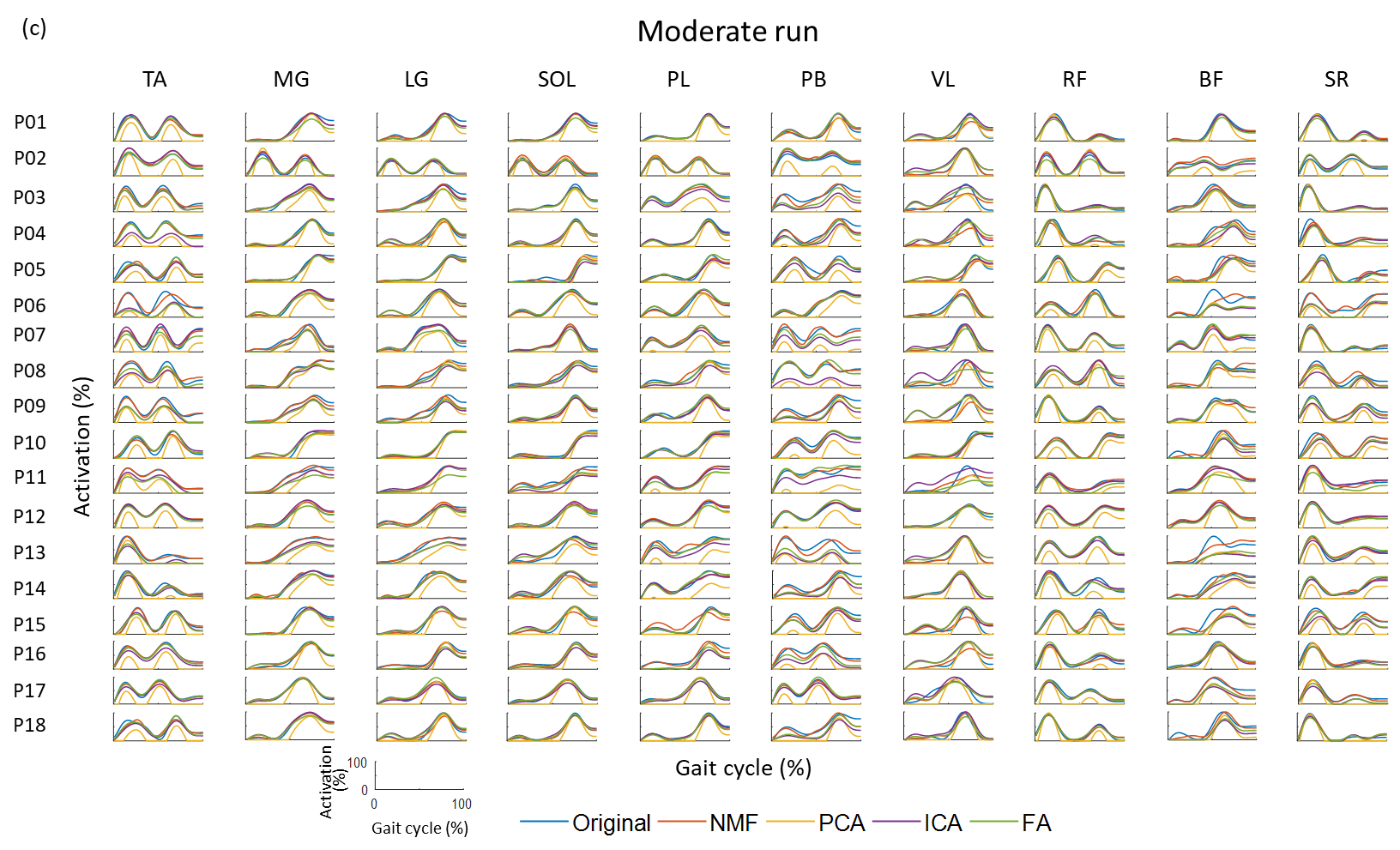


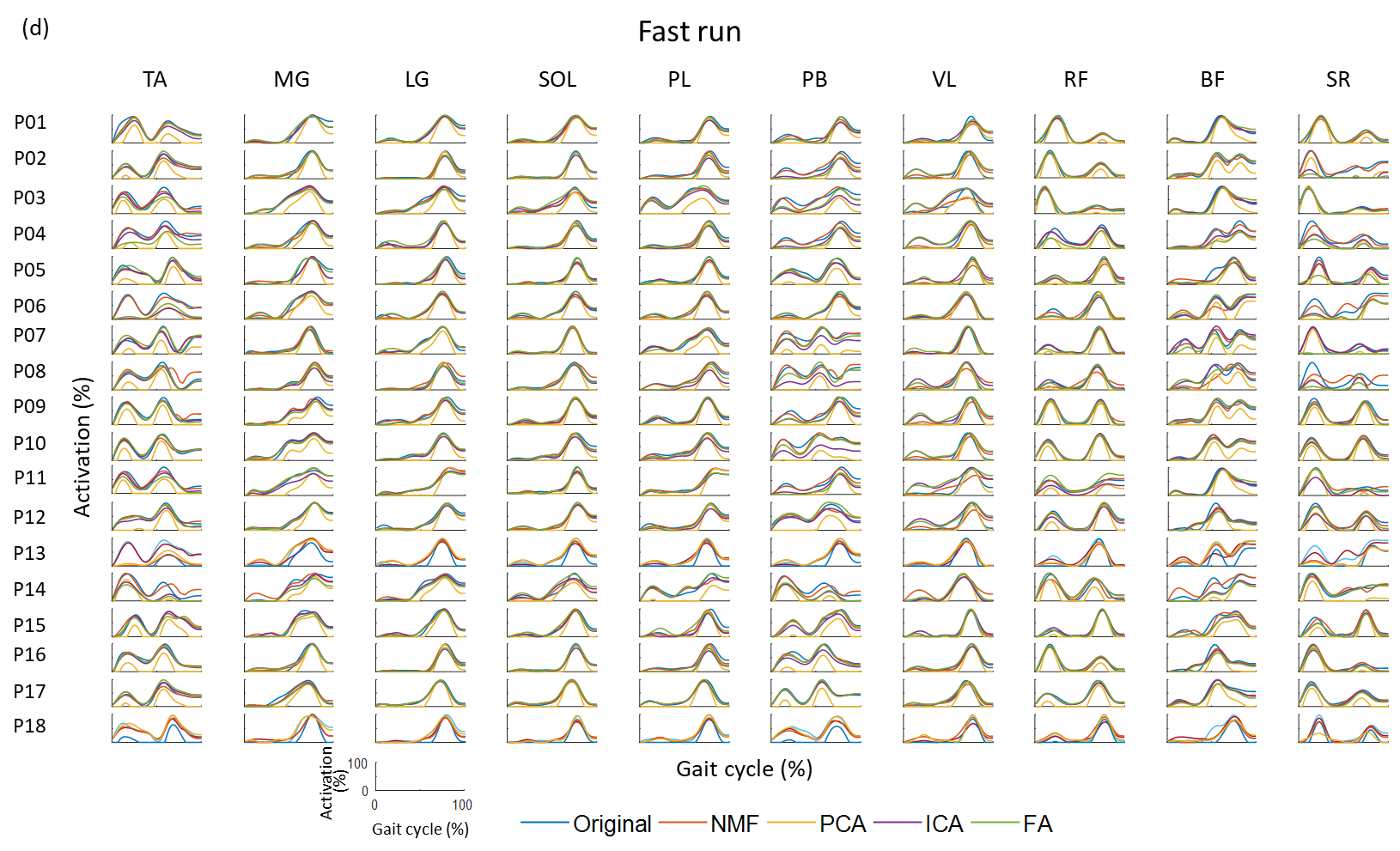


**Supplementary Figure S3.** Original and reconstructed muscle activation patterns from 10 muscles of 18 subjects during (a) walking, and (b) slow, (c) moderate, and (d) fast running conditions.

**Probability density function of synergy excitation primitives**


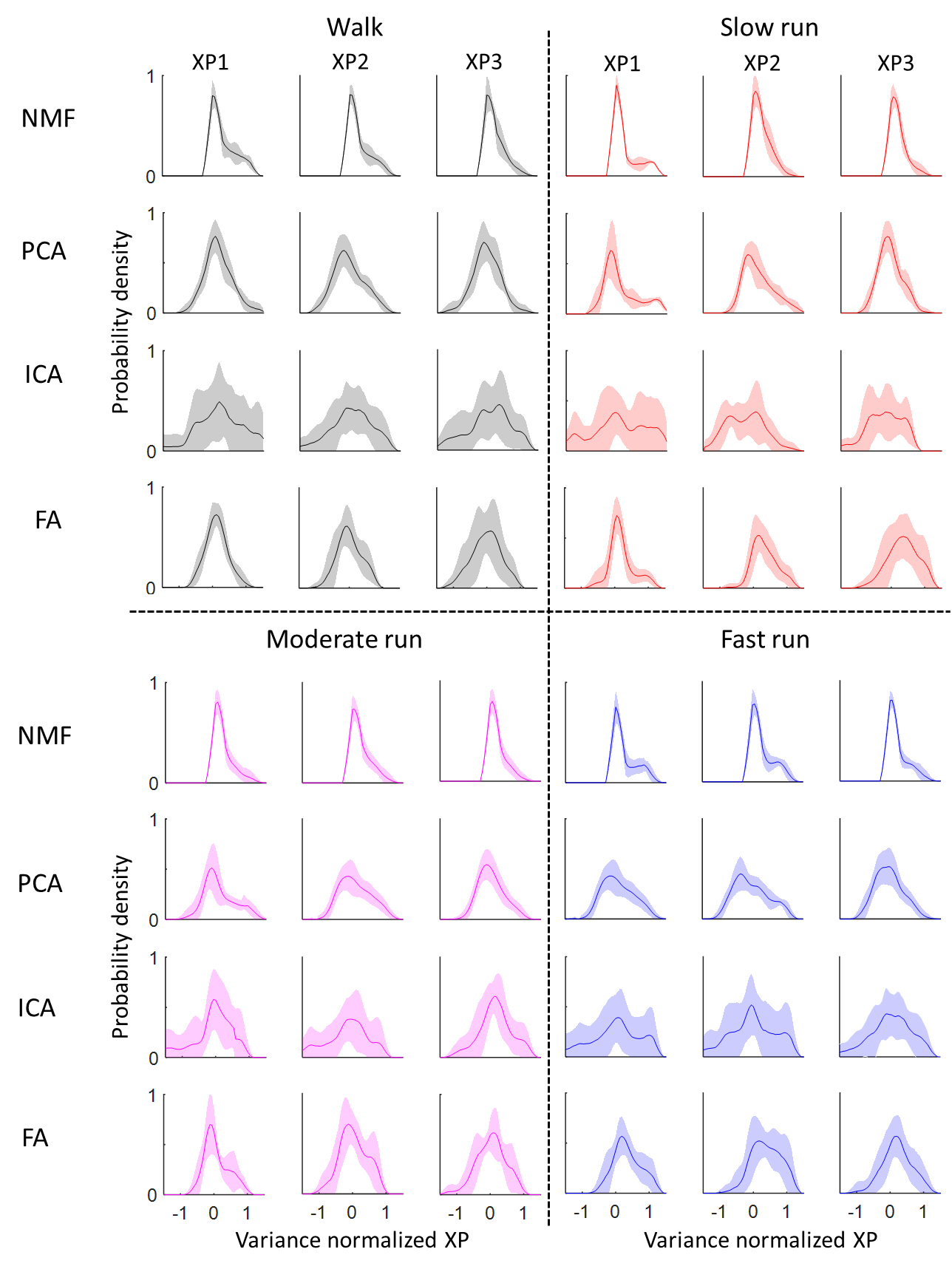


**Supplementary Figure S4.** Probability distribution of three synergy excitation primitives from four factorisation methods for all participants during walking and slow, moderate, and fast running conditions. Shaded regions indicate standard deviation across participants.

**Empirical cumulative distribution of muscle synergy excitation primitives**


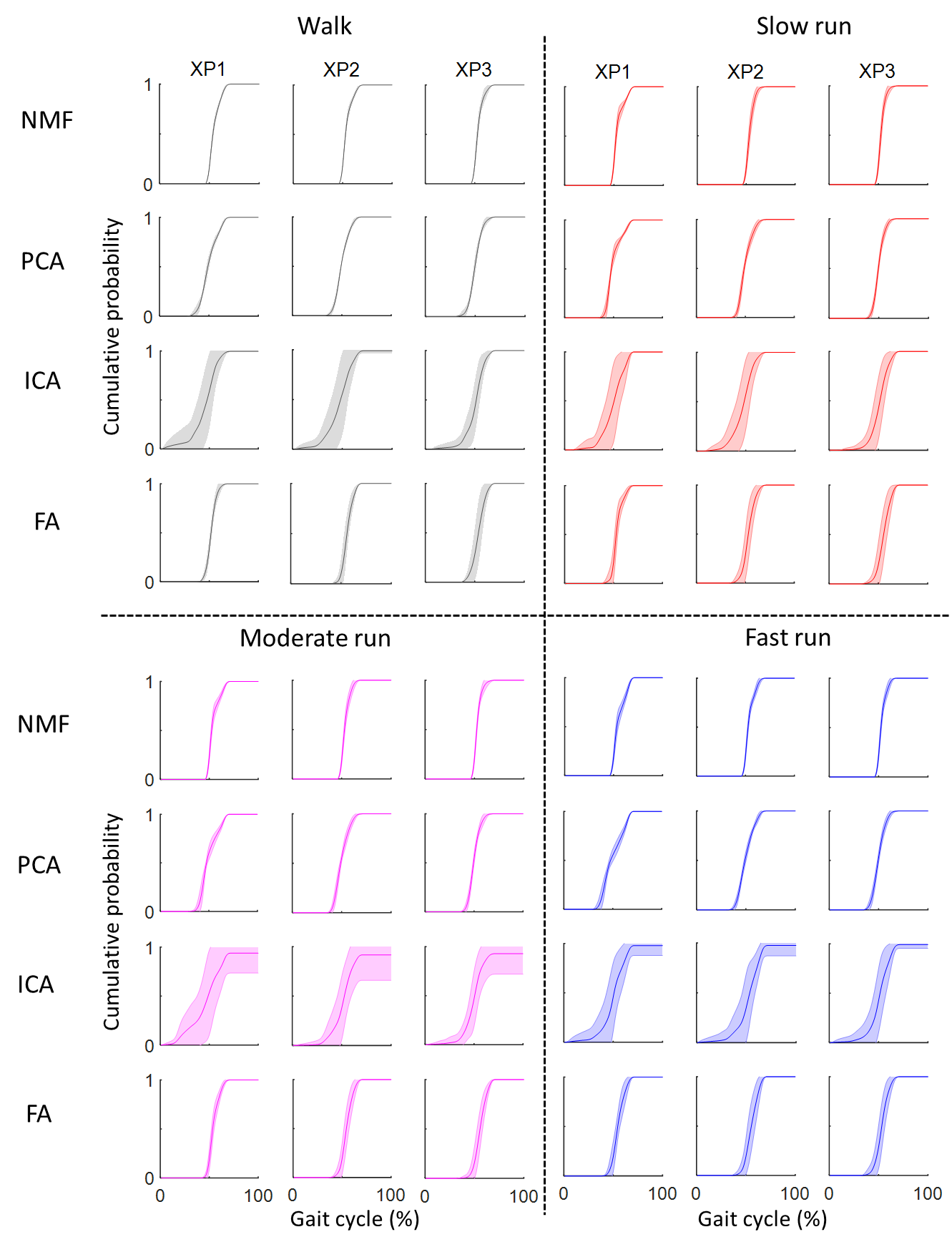


**Supplementary Figure S5.** Empirical cumulative distribution of three synergy excitation primitives from four factorisation methods for all participants during walking and slow, moderate, and fast running conditions. Shaded regions indicate standard deviation across participants.

**Probability density function of linear enveloped EMG data**


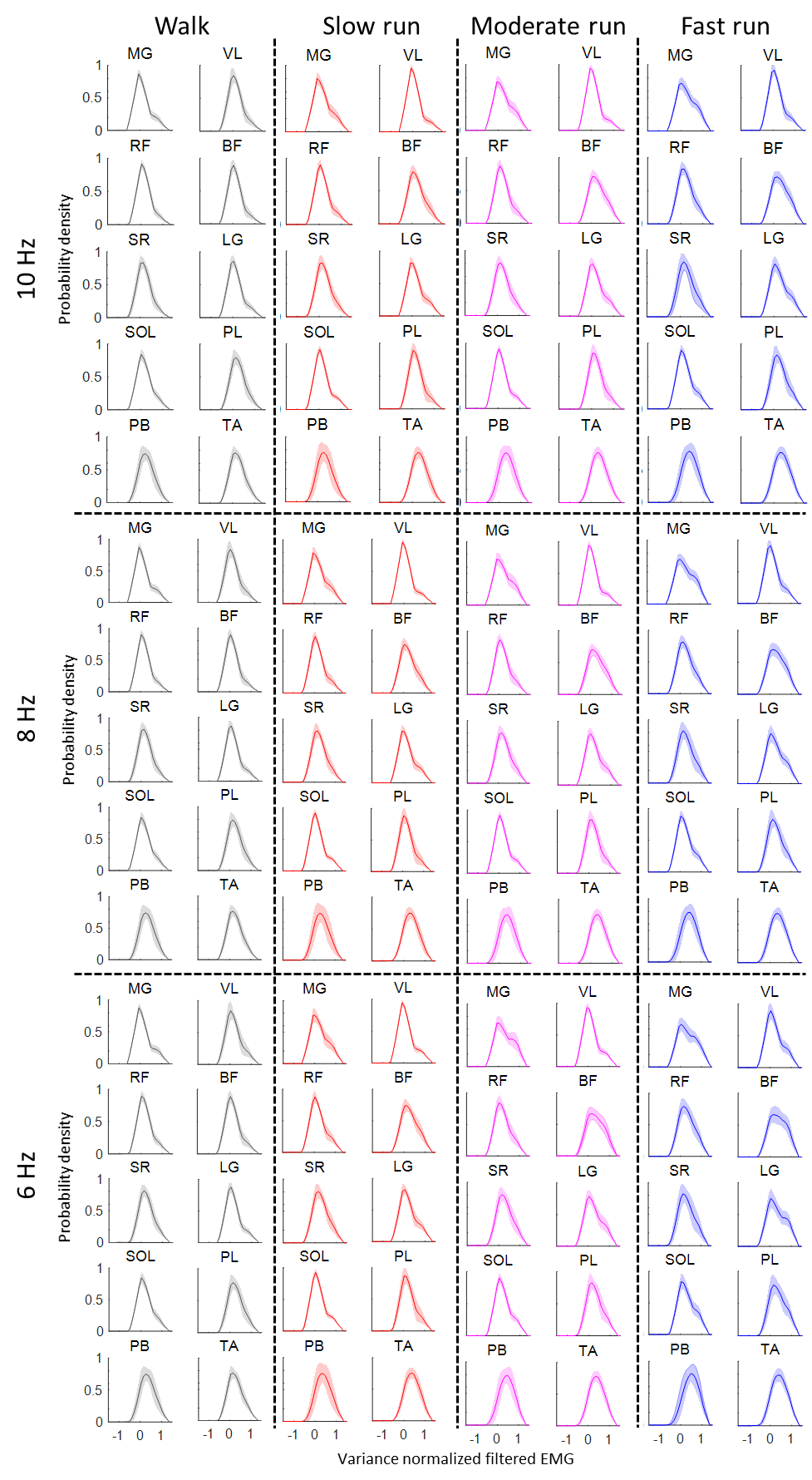


**Supplementary Figure S6.** Probability density function of EMG data filtered at three different cut-off frequencies (top: 10 Hz, middle: 8 Hz; bottom: 6 Hz) for all muscles across four gait speeds.

**VAF, occurrence of agreement and maximum dissimilarity**

**
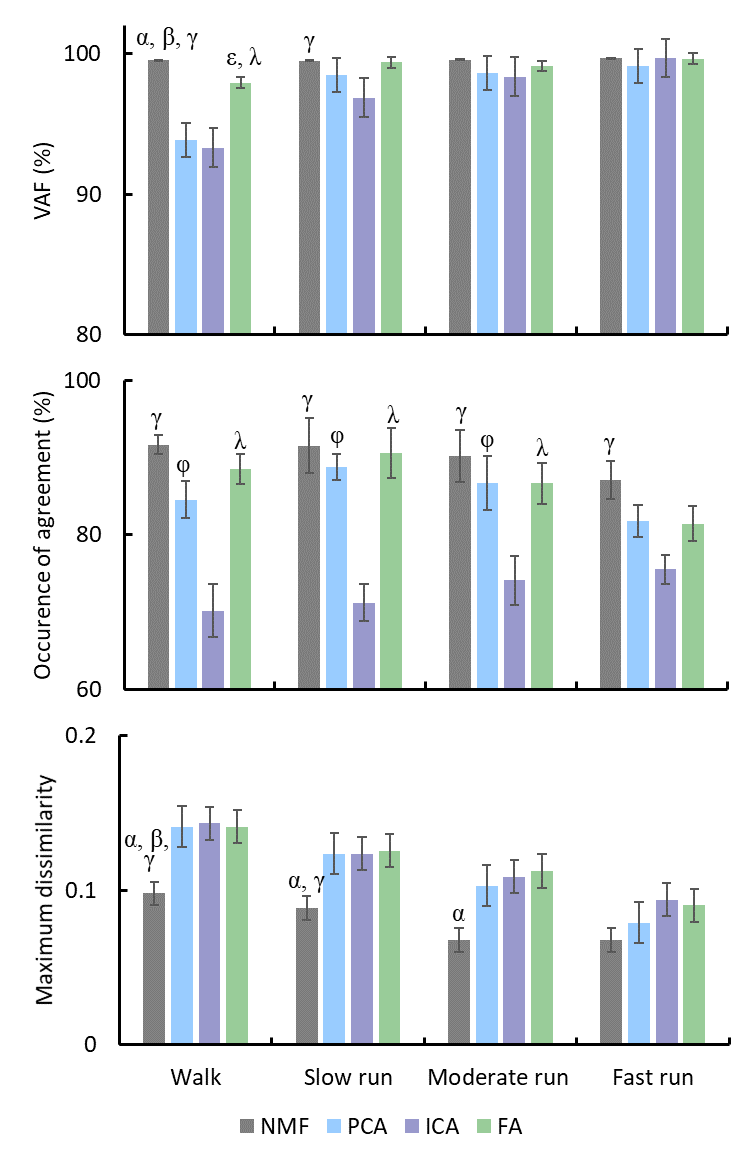
**

**Supplementary Figure S7.** (a) VAF (%), (b) occurrence of agreement (%), and (c) maximum dissimilarity between the cumulative distribution function (ECDF) of the muscle activation patterns and synergy excitation primitives at four gait speeds. Error bars indicate standard deviation. The following symbols represent significant differences (p < 0.05) between factorisation methods, α: NMF > FA or NMF < FA, β: NMF > PCA, γ: NMF > ICA or NMF < ICA, ε: FA > PCA, λ: FA > ICA, ρ: ICA > PCA, φ: PCA > ICA.

**Supplementary Table T1.** Average (± standard deviation) kurtosis and skewness of the synergy excitation primitives from each factorisation method during walking and running at different speeds (10 trials each).

| Kurtosis |  | Walk | Slow run | Moderate run | Fast run |
| --- | --- | --- | --- | --- | --- |
|  | NMF | 3.15(±0.60) | 2.98(±0.40) | 2.78(±0.37) | 2.40(±-.32) |
|  | PCA | 2.70(±0.40) | 2.72(±0.38) | 2.39(±0.37) | 2.07(±0.25) |
|  | ICA | 2.61(±0.70) | 2.05(±0.77) | 1.81(±0.25) | 1.62(±0.2) |
|  | FA | 1.50(±4.5e-3) | 1.50(±2.8e-3) | 1.49(±3.9e-3) | 1.49(±2.1e-3) |
|  |  |  |  |  |  |
| Skewness | NMF | 1.09(±0.20) | 0.98(±0.22) | 0.97(±0.22) | 0.92(±0.21) |
|  | PCA | 0.48(±0.30) | 0.64(±0.24) | 0.50(±0.28) | 0.37(±0.23) |
|  | ICA | 0.02(±0.65) | -0.003(±0.32) | -0.02(±0.21) | -0.01(±0.10) |
|  | FA | 0.03(±0.16) | 0.04(±0.16) | 0.03(±0.15) | 0.02(±0.15) |

FA: factor analysis; ICA: independent component analysis; NMF: non-negative matrix factorisation; PCA: principal component analysis.
